# AI-Driven Rapid Screening and Characterization of Dipeptidyl Peptidase-IV (DPP-IV) Inhibitory Peptides from Goat Blood Proteins: An Integrative *In Silico* and Experimental Strategy

**DOI:** 10.3390/foods15020398

**Published:** 2026-01-22

**Authors:** Jingjie Tan, Sirong Huang, Dongjing Wu, Zhongquan Zhao, Yongju Zhao, Yu Fu, Wei Wu

**Affiliations:** 1 Chongqing Key Laboratory of Herbivore Science, College of Animal Science and Technology, Southwest University, Chongqing 400715, China; 2 College of Food Science, Southwest University, Chongqing 400715, China

**Keywords:** blood protein, DPP-IV inhibitory peptides, machine learning, molecular docking, molecular dynamics

## Abstract

To enhance the screening efficiency of bioactive peptides, an AI-driven approach was employed to screen DPP-IV inhibitory peptides from goat blood proteins by an integrated *in silico*, *in vitro*, and machine learning strategy. Furthermore, the inhibitory mechanism of DPP-IV inhibitory peptides was elucidated by kinetics, molecular docking and simulation. Additionally, their *in vitro* digestive stability was assessed. *In silico* results revealed that goat blood proteins were promising precursors of DPP-IV inhibitory peptides, while bromelain was the optimal protease. Their peptide sequences were further identified by peptidomics and predicted by self-developed machine learning models (LightGBM) to identify the potent DPP-IV inhibitory peptides. Two novel DPP-IV inhibitory peptides were identified (FPL and FPHFDL). Enzyme kinetics, molecular docking and molecular simulation data indicated that FPL served as a competitive inhibitor, whereas FPHFDL was a non-competitive inhibitor. Overall, the integrative *in silico* and *in vitro* strategy is feasible for rapid screening of DPP-IV inhibitory peptides from goat blood proteins.

## 1. Introduction

Blood, a by-product of goat slaughtering, is abundant in high-quality protein, but has long been underutilized. Goat blood contains 17–22% protein, primarily composed of hemoglobin (60–70%) and plasma proteins. Plasma consists of 50–60% albumin, 40–50% globulin, and a small amount of fibrinogen [1]. Hemoglobin is a heterotetrameric protein (α2β2) that contains a heme group with an iron atom in each subunit. The presence of bound iron results in a dark red color of hemoglobin and imparts a metallic taste during processing, which significantly limits its utilization [2]. Furthermore, functional protein components in goat blood, such as superoxide dismutase and thrombin. Currently, goat blood resources are mostly utilized in low-value applications (e.g., animal feed, fertilizer, or direct disposal), leading to significant resource waste and environmental pollution.

In recent years, the preparation of food-derived bioactive peptides through enzymatic hydrolysis has become an important strategy for enhancing the value of animal by-products [3]. Bioactive peptides, released during enzymatic hydrolysis, can exert various biological functions such as antioxidant, antibacterial, antihypertensive, antidiabetic, and anti-obesity activities [4,5]. Peptides with antioxidant, antihypertensive, antihypertensive, and antibacterial activities have been found in porcine and sheep blood protein [6,7].

Diabetes has become one of the most prevalent chronic diseases globally, with its incidence continuing to rise annually. According to the 11th edition of the IDF Diabetes Atlas, the prevalence of diabetes was 11.1% in 2025. It is estimated to rise to 13% by 2050 [8]. Type 2 diabetes mellitus (T2DM) is one of the most common types, accounting for over 90% of cases. Currently, clinical practice primarily relies on synthetic DPP-IV inhibitors (e.g., sitagliptin) to regulate blood glucose by prolonging half-life of incretins (GLP-I and GIP). However, long-term use of these inhibitors can lead to side effects, such as nasopharyngitis and inflammatory bowel disease [9]. Therefore, screening for safe DPP-IV inhibitory peptides from natural proteins has become an important direction in the field of food science.

*In silico* methods provide robust support for screening bioactive peptides, significantly enhancing both screening efficiency and accuracy. Compared to traditional screening methods, the primary advantages include: (1) Activity prediction. Based on the BIOPEP-UWM database, the bioactivity of proteins from different sources can be evaluated [10]. (2) Enzymatic hydrolysis optimization. The enzymatic hydrolysis of proteins can be assessed under the action of different enzymes (e.g., pepsin, bromelain). And the release of bioactive peptides can be guided [11]. (3) Efficient screening. Machine learning has emerged as an effective paradigm for high-throughput discovery of bioactive peptides [12,13]. By integrating large-scale historical datasets with optimized algorithmic frameworks, this approach systematically deciphers the structure–activity relationships between peptide sequences and biological functions, thereby enabling accurate prediction of peptide bioactivities. While PeptideRanker, a well-established machine learning model, demonstrates broad-spectrum bioactivity prediction capabilities, its performance in predicting specific activity types, such as DPP-IV inhibitory potency, remains constrained [14]. (4) Structure prediction. Molecular docking helps determine the binding cavity of peptides and proteins, while molecular dynamics simulations elucidate their binding sites and inhibition mechanisms under specific force fields [15]. *In silico* analysis not only significantly accelerates the research and development efficiency of bioactive peptides but also reduces associated research costs.

Goat blood serves as a valuable precursor for bioactive peptides. However, its potential, particularly regarding DPP-IV inhibitory peptides critical for treating T2DM, remains underutilized. Traditional enzymatic hydrolysis methods present challenges, including time-consuming preparation. Therefore, this study employed an *in silico* approach to predict the bioactivity potential of goat blood proteins and identify the optimal protease. DPP-IV inhibitory peptides were released from goat blood proteins by *in vitro* enzymatic hydrolysis, and peptidomics was utilized to characterize the peptides effectively. An efficient and reliable classification model was constructed, based on machine learning, which was used to predict the activity of goat blood peptides. In the process of model construction, the feature importance analysis was carried out, which helped to explain the characteristics of DPP-IV inhibitory peptides. Additionally, the mechanisms of action and digestive stability were investigated. This study provides a theoretical reference for rapid screening of effective DPP-IV inhibitory peptides.

## 2. Materials and Methods

### 2.1. Materials

Goat blood powder (protein content, 88.2%) was purchased from Shandong Huachen Biotechnology Co., Ltd. (Shandong, China); Pepsin 1:10,000 (1403GR005, 10,000 U/g) and Trypsin 1:250 (1004GR025, 250 U/g) were purchased from Guangzhou Saiguo Biotechnology Co., Ltd. (Guangzhou, China). Bromelain (600 U/g) and recombinant human DPP-IV (purity > 95%) were purchased from Beijing Solarbio Science & Technology Co., Ltd. (Beijing, China). Chymotrypsin (activity ≥ 1000 USP U/mg) was purchased from Sangon Biotech Co., Ltd. (Shanghai, China). Tris-HCl buffer (purity, 99%) was purchased from Shanghai Macklin Biochemical Technology Co., Ltd. (Shanghai, China). Gly-Pro-p-nitroanilide hydrochloride (purity, 97%) and Alogliptin (purity > 99%) were purchased from Aladdin Biochemical Technology Co., Ltd. (Shanghai, China). FPL and FPHFDL (purity > 98%) were chemically synthesized by Sangon Biotech Co., Ltd. (Shanghai, China). All other reagents used in this study were of analytical grade.

### 2.2. In Silico Analysis

*In silico* hydrolysis analysis was performed according to a previous study [16]. The amino acid sequences of five major proteins in goat blood (hemoglobin subunit alpha1, hemoglobin subunit alpha2, hemoglobin subunit beta-A, hemoglobin subunit beta-C, and albumin) were obtained from the UniProt database (http://www.uniprot.org, accessed on 1 June 2024). The complete sequence information is provided in Table S1. The bioactivity index (*A* value) of protein was computed, utilizing the “Calculation” feature within the BIOPEP database (https://biochemia.uwm.edu.pl/biopep/start_biopep.php, accessed on 1 June 2024). The *A* value represents the frequency of bioactive peptide occurrence within a protein sequence, calculated as follows.

A=a/N

where *A* denotes the frequency of bioactive peptide occurrence in the protein sequence; *a* represents the number of peptides exhibiting specific bioactivity and *N* signifies the total number of amino acid residues in the protein. A higher *A* value suggests a greater potential for the protein to serve as a precursor for bioactive peptides. However, *A* value does not account for the inhibitory activity potency of released peptides. Further validation through *in vitro* experiments is necessary. Using the “Enzyme action” function of the BIOPEP database, *in silico* simulated hydrolysis of goat blood protein was performed, employing five classes of 30 proteases. A complete list of enzymes is provided in Table S2.

### 2.3. Determination of Protein Content and Amino Acid Composition of Goat Blood

The protein content of goat blood meal was determined according to a previous study using the Kjeldahl method [17]. Goat blood meal (0.3 g), copper sulfate (0.4 g), and potassium sulfate (6.0 g) were weighed into a digestion tube, followed by the addition of 20 mL of sulfuric acid solution, and subsequently placed in a digestion furnace. Following digestion, the mixture was cooled, and water (50 mL) was added. Subsequently, the solution underwent liquid addition, distillation, and titration using an automatic Kjeldahl nitrogen analyzer (Model K1160, Haineng Future Technology Group Co., Ltd., Jinan, China), and the data were recorded. The protein content was calculated using the following formula:

$$X\;\left(\frac{g}{100g}\right)=\frac{\left(V_{1}-V_{2}\right)}{m\times \frac{V}{100}}\times c\times 0.014\times F\times 100$$

where V_1_ represents the volume of standard sulfuric acid solution consumed by the sample during titration (mL); V_2_ represents the volume of standard sulfuric acid solution consumed by the blank reagent during titration (mL); c is 0.1 M; m is the mass of the sample (g); V is the volume of the digestion solution taken (mL); F is 5.7.

Amino acid composition analysis of goat blood meal was performed using an automatic amino acid analyzer (Hitachi, L8900, Tokyo, Japan). Goat blood meal (0.2 g) was hydrolyzed with 6 M HCl at 110 °C for 24 h. Hydrolysate was dissolved in a sodium citrate buffer solution and transferred to an injection vial for determination using the automatic amino acid analyzer (Hitachi, L8900, Tokyo, Japan), according to our previously reported method [18].

### 2.4. Preparation of DPP-IV Inhibitory Peptides from Goat Blood Protein

According to the *in silico* analysis, goat blood hydrolyzed by bromelain may generate more DPP-IV inhibitory peptides. The method was as follows. Goat blood meal was dissolved in water at a ratio of 1:10 (*w*/*v*), with an enzyme-to-substrate ratio (E/S, *w*/*w*) of 1:5. Enzymatic hydrolysis was performed under the optimal conditions of bromelain (pH 6.4, 55 °C) for 7 h. During the enzymatic hydrolysis, 10 mL of the hydrolysate was taken every 1 h and placed in boiling water to inactivate the enzyme for 15 min. After cooling to room temperature, the pH was adjusted to 4.0 to dissociate the heme groups, and the mixture was centrifuged at 10,000× *g* for 20 min. The supernatant was collected, and the pH was adjusted to 7.0 before being lyophilized for subsequent use.

### 2.5. Degree of Hydrolysis (DH)

The DH was determined using a modified OPA method [16]. 10 µL of the hydrolysate was mixed with 1.2 mL of OPA reagent at room temperature, protected from light for 10 min. Then, the absorbance was measured at 340 nm using a visible light spectrophotometer (Macy, V-1500, Shanghai, China). The concentration of free amino groups and the DH were calculated using Leu standard curve.

$$DH\left(\%\right)=\frac{\left[{NH}_{2}\right]\times V_{1}\times m}{{\left[{NH}_{2}\right]}_{tot}\times V\times m_{1}}\times 100\%$$

where [NH_2_] is the concentration of free amino groups in the hydrolysate (M); V_1_ is the volume of the sample hydrolysate (mL); m_1_ is the mass of the sample (g); [NH_2_]_tot_ is the total concentration of free amino groups in the completely hydrolyzed sample (M); V is the final volume of the completely hydrolyzed sample solution (mL); and m is the mass of the completely hydrolyzed sample (g).

### 2.6. Measurement of Molecular Weight (MW) Distribution

The MW distribution was determined according to a previously reported using size-exclusion chromatography (SEC). The determination conditions were as follows. Chromatographic column: BioBasic SEC120 (7.8 × 300 mm, 5 µm). Mobile phase is acetonitrile: water: trifluoroacetic acid = 30:70:0.1; detection wavelength: 214 nm; column temperature: 25 °C; flow rate: 0.5 mL/min; injection volume: 20 µL. The standards and samples were diluted with deionized water to a 1 mg/mL solution and filtered through a 0.22 µm filter membrane for MW determination. MW standards included Gly-Gly-Tyr-Arg (451.48 Da), aprotinin (6511 Da), cytochrome C (12,500 Da), and human serum albumin (66,000 Da). A standard curve was plotted with the standard MW logarithm as the ordinate and the corresponding retention time (RT) as the abscissa. The MW of the sample was calculated according to the MW standard curve equation.

### 2.7. Evaluation of DPP-IV Inhibitory Activity

The *in vitro* hypoglycemic activity of protein hydrolysates is typically assessed using DPP-IV inhibition activity. The DPP-IV inhibition assay was performed according to a previous method [19] with slight modifications. A mixture of DPP-IV (2 µg/mL, 40 µL) and hydrolysate (80 µL) was prepared in a 96-well plate. Alogliptin at 295 nM was used as the positive control and Tris-HCl was used as the control group. The mixture was incubated at 37 °C for 10 min, followed by the addition of Gly-Pro-pNA (0.5 mM, 80 µL) and vortexed for 30 s. The reaction was performed at 37 °C for 30 min in a microplate reader, with absorbance measured at 405 nm at 1 min interval. The DPP-IV inhibitory activity was calculated as follows:

$$DPP\text{-}IV\;inhibition\;\left(\%\right)=\left(1-\frac{{slope}_{sample}}{{slope}_{control}}\right)\times 100\%$$

where ${slope}_{control}$ represents the slope of the time-absorbance curve for the control group; ${slope}_{sample}$ represents the slope of the time-absorbance curve for the experimental group.

### 2.8. Identification and Screening of Peptides Based on Peptidomics

DPP-IV inhibitory peptides were identified by LC-MS/MS technology with database searching and de novo sequencing. The conditions were as follows. Sample pretreatment. 200 µg of freeze-dried sample was reconstituted in 0.1% TFA/H_2_O and ultrafiltered (ultrafiltration membrane: 10 kDa). The filtrate was collected and desalted using C18 desalting column, followed by activation and column equilibration with 200 µL methanol and 200 µL 0.1% TFA/ddH_2_O (*v*/*v*). The column was washed with 200 µL 0.1% TFA/ddH_2_O, and peptides were eluted with 200 µL, 80% acetonitrile/0.1% TFA. The eluate was collected and lyophilized for subsequent analysis. LC-MS/MS analysis was performed using high-performance liquid chromatograph (Dionex U300, Thermo Fisher Scientific, Waltham, MA, USA); the chromatographic column was C18, 3 µm, 100 Å, 75 µm × 15 cm; mobile phase A: 0.1% formic acid in water; B: 0.1% formic acid in acetonitrile; mass spectrometer: Thermo Scientific Q Exactive HFX (Waltham, MA, USA); parameter settings: spray voltage of 4.1 kV; capillary temperature: 320 °C; S-lens RF Level: 60; resolution settings: MS1 120,000 (m/z 200), MS2 15,000 (m/z 200); precursor ion scan range: m/z 340–1100; product ion scan range: m/z 110; MS1 AGC: 3 × 10^6^, ion injection time: 20 ms; MS2 AGC: 2 × 10^4^, ion injection time: 30 ms; isolation window: 1.6 m/z; fragmentation mode: HCD, energy NCE 27; Data-dependent MS/MS: Top 25; dynamic exclusion: 15 s; charge exclusion was set for 7, 8 and >8. Data processing. The LC-MS/MS spectra were first subjected to de novo sequencing by Peaks Studio 12.5 (Bioinformatics Solutions Inc., Waterloo, ON, Canada). The resulting peptides were filtered, retaining only those with an Average Local Confidence (ALC) score ≥ 50%. Then, the de novo sequences were used as queries to search against the protein database, downloaded from NCBI (retrieved 30 May 2024, Species: *Capra hircus*, ID:9925), with the confidence threshold set at −10lgP ≥ 15. For short peptide sequences (typically hexapeptides or shorter) that could not be matched in the protein database, BLAST analysis (https://blast.ncbi.nlm.nih.gov/Blast.cgi; accessed on 10 November 2024) was employed to perform homology search against broader protein databases to explore their potential origins and exclude sequences from non-target sources. PeptideRanker (http://distilldeep.ucd.ie/PeptideRanker/; accessed on 10 November 2024) was applied to evaluate the bioactive potential of peptides [20].

### 2.9. Machine Learning Model Training

#### 2.9.1. Dataset Construction

DPP-IV inhibitory peptide sequences and their *IC*_50_ values were obtained from the BIOPEP database (https://biochemia.uwm.edu.pl/biopep/start_biopep.php; accessed on 5 July 2025), DFBP database (http://www.cqudfbp.net/index.jsp; accessed on 5 July 2025), and the relevant literature. Considering the distribution of compound activities, an activity threshold of 2000 µM was set: peptides with *IC*_50_ ≥ 2000 µM were labeled as inactive (tag “0”), while those with *IC*_50_ < 2000 µM were labeled as active (tag “1”). The final dataset comprised 588 active peptides and 210 inactive peptides. Features were extracted using the pseudo amino acid composition (PseAAC) method [21]. Each peptide sequence was transformed into a 27-dimensional feature vector, including the relative frequencies of 20 standard amino acids, one hydrophobicity-related factor, and six physicochemical properties (Hydrophobicity, Hydrophilicity, Mass, pK_1_, pK_2_, pI).

#### 2.9.2. Machine Learning Models

Based on the method by Zhang et al. [22], four machine learning algorithms were employed to construct classification models, namely GBDT, RF, LightGBM and XGBoost. During the training phase, hyperparameters were optimized via grid search combined with 5-fold cross-validation. The 5-fold cross-validation involved partitioning the dataset into five equal subsets, iteratively training on four subsets and validating on the remaining one, thereby providing a comprehensive assessment of model performance across different data splits. This approach enhances the evaluation of the model’s generalization capability. Grid search traversed the predefined hyperparameter space to identify the optimal configuration, with the area under the receiver operating characteristic curve (AUC) serving as the primary evaluation metric, supplemented by accuracy comparisons.

Model performance was comprehensively evaluated using multidimensional metrics [23], including accuracy (the proportion of correctly predicted samples), recall (the proportion of true positives correctly identified), precision (the proportion of predicted positives that are true positives), F1-score (the harmonic mean of precision and recall), and ROC-AUC (the area under the ROC curve, quantifying the model’s ability to distinguish between positive and negative samples across various thresholds; range [0, 1], with values closer to 1 indicating superior performance).

#### 2.9.3. Test Set Prediction

Independent test set prediction was executed in three stages. First, sequences from the test set were subjected to feature extraction using the identical PseAAC method, followed by feature transformation with the standardizer preserved during the training phase. Subsequently, the optimal model was loaded to output predicted labels and probabilities. Finally, high-confidence candidate inhibitors were screened as candidate molecules for experimental validation. All computations were implemented in Python 3.10, utilizing key libraries including pandas, numpy, scikit-learn, LightGBM, XGBoost, and joblib, under the Windows 10 operating system.

### 2.10. Synthesis and Characterization of Peptides

Both FPL and FPHFDL were synthesized by Sangon Biotech Co., Ltd. (Shanghai, China) using standard solid-phase peptide synthesis. The purity of FPL was determined by HPLC (C18 column, detection at 214 nm) to be 99.35%, and its molecular weight was confirmed by LC-MS/MS (375.25 Da). The purity of FPHFDL was determined by HPLC (C18 column, detection at 214 nm) to be 98.66%, and its molecular weight was verified by LC-MS/MS (744.70 Da). Both synthetic peptides were supplied as lyophilized powders, stored at −20 °C, and dissolved in 100 mM Tris-HCl buffer (pH 8.0) for further use.

### 2.11. Enzyme Inhibition Kinetics

The enzyme inhibition kinetics of DPP-IV by synthetic peptides were analyzed following the method in Section 2.7. Different concentrations of synthetic peptide (0–800 µM) were mixed with varying substrate concentrations (0.025–0.4 µM), and the initial velocity of the reaction (V_i_) was determined in the resulting mixtures. The data were analyzed by nonlinear regression, using GraphPad Prism software 10.1.2 to calculate the *K_m_* and *V_max_*. Lineweaver–Burk plots were plotted using 1/[S] (reciprocal of substrate concentration) as the abscissa and 1/V_i_ (reciprocal of the initial velocity of the reaction) as the ordinate. The equations are listed below.

$$\frac{1}{v_{i}}=\frac{K_{m}}{V_{max}}\left(1+\frac{\left[I\right]}{K_{i}}\right)\frac{1}{\left[S\right]}+\frac{1}{V_{max}}$$

The inhibition constant (*K_i_*) was calculated by the following equation:

Competitive inhibitor: $K_{m}^{app}=K_{m}\times (1+\frac{\left[I\right]}{K_{i}})$

Non-competitive inhibitor: $V_{max}^{app}=\frac{V_{max}}{1+\frac{\left[I\right]}{K_{i}}}$

Where $K_{m}^{app}$ is the apparent *K_m_*; $V_{max}^{app}$ is the apparent *V_max_*; *K_i_* is the dissociation constant of the inhibitor. The inhibitory type of synthetic peptides on DPP-IV was further determined.

### 2.12. Molecular Docking

Semi-flexible molecular docking was used to predict the binding mode of synthetic peptide to DPP-IV. Docking analysis was performed using Autodock vina 1.2.0 [24]. The crystal structure of DPP-IV (PDB ID: 1WCY) was obtained from PDB database (https://www.rcsb.org/; accessed on 1 December 2024) as receptor protein. The 3D structures of synthetic peptides were plotted using Avogadro 1.2.0 as ligand.

Prior to docking, receptor protein was pre-processed using PYMOL software 3.1.6.1. Water molecules were removed, hydrogen atoms were added, and redundant small molecules (ligands, ions, etc.) unrelated to the main chain were deleted. The PDBQT file was generated in AutoDock Tools 1.5.7 [25] for subsequent docking. Ligands were pre-processed in AutoDock Tools by adding charges, setting rotatable bonds, and generating ligand PDBQT files. Molecular docking was performed using AutoDock Vina 1.2.7. Docking box was set to cover the entire receptor protein, and simulated docking was performed 100 times to increase the reliability of the results. The structural conformation with the lowest Vina score was then output as the correct one. Docking results were visualized using LigPlot+2.2.9 [26] to visualize the interaction pattern of peptide with DPP-IV and to analyze the role of key amino acid residues at 2D levels.

### 2.13. Molecular Dynamics Simulations

Molecular dynamics simulations were performed based on the ligand–receptor complex obtained by docking as the initial structure. Meanwhile, empty protein was used as a control to explore the changing trend of the complex between DPP-IV and synthetic peptide molecules before and after docking. The simulations were performed using AMBER 18 software [27]. Prior to simulation, the charge of ligand was calculated by antechamber module and Hartree-Fock (HF) SCF/6-31G* of Gaussian 09 software. Molecular mechanics parameters from the GAFF2 force field and ff14SB force field were, respectively, assigned to ligand and receptor [28]. The LEaP module was used for each system to add hydrogen atoms. The structures were solvated in a rectangular box of TIP3P water, with the extended range of 10 Å. Na^+^/Cl^−^ was added to the system to balance the system charge.

Molecular dynamics simulations were performed using AMBER18 software package. Prior to simulations, energy minimization of system was conducted, including 2500 steps of steepest descent and 2500 steps of conjugate gradient methods. Following energy minimization, the system was heated from 0 K to 298.15 K over 200 ps. Subsequently, 500 ps NVT (isothermal-isochoric) ensemble simulation was performed at 298.15 K to allow for uniform distribution of solvent molecules within the solvent box. Finally, 500 ps equilibration simulation was conducted under NPT (isothermal-isobaric) conditions. Ultimately, two complex systems underwent 100 ns NPT ensemble simulations under periodic boundary conditions. During the simulations, Particle Mesh Ewald (PME) method was utilized for long-range electrostatic interactions (cutoff = 10 Å), and SHAKE algorithm was used to constrain hydrogen bond lengths. The Langevin algorithm was used for temperature control, with a collision frequency γ set to 2 ps-1 [29]. The system pressure was maintained at 1 atm, with an integration time step of 2 fs. Trajectories were saved every 10 ps for subsequent analysis, including protein–ligand complex root mean square deviation (RMSD), root mean square fluctuation (RMSF), radius of gyration (RoG) and solvent accessible surface area (SASA).

MMGBSA binding free energy calculations. The binding free energy was investigated using the MM-GBSA method [30]. In this study, 90–100 ns MD trajectories were used for the calculations, according to the following equation:

$$∆G_{bind}=∆G_{complex}-\left(∆G_{receptor}+∆G_{ligand}\right)\;=∆E_{internal}+∆E_{VDW}+∆E_{elec}+∆G_{GB}+∆G_{SA}$$

where Δ*E*_internal_ represents internal energy; Δ*E*_VDW_ represents van der Waals interactions; Δ*E*_elec_ represents electrostatic interactions. Δ*G*_GB_ and Δ*G*_SA_ collectively represent the solvation free energy. *G*_GB_ is the polar solvation-free energy, and *G_SA_* is the nonpolar solvation-free energy. The GB model developed by a previous study [31], where (igb = 2) was used for calculations Δ*G*_GB_. The nonpolar solvation-free energy (Δ*G*_SA_) was calculated based on the product of surface tension (γ) and the solvent-accessible surface area (SA), Δ*G*_SA_ = 0.0072 × Δ*SASA*. The entropy change was omitted in this study due to high computational resource requirements and low accuracy.

### 2.14. Gastrointestinal Digestive Stability

According to the protocol of INFOGEST 2.0 [32], the simulated gastrointestinal digestion was performed, and the *IC*_50_ of DPP-IV before and after digestion was measured to assess the stability of synthetic peptides.

#### 2.14.1. Enzyme Activity Assay

Initially, the activities of enzymes used (pepsin, trypsin, and chymotrypsin) were determined. The method was listed as follows. Azocasein (21.185 µM, 125 µL) was mixed with an equal volume of enzyme solution (100 µg/mL), and the mixture was vortexed. The mixture solution was oscillated at 300 rpm for 30 min, 750 µL of trichloroacetic acid (TCA) was added to terminate the reaction. The mixture was further centrifuged at 3000 rpm for 7 min, and 125 µL of the supernatant was transferred to a 96-well plate. Subsequently, NaOH (0.5 M, 125 µL) was added, and the absorbance was measured at 410 nm using a microplate reader. Standard curves were generated by incubating 0–80 µM azocasein with bromelain (1 mg/mL) for 7 h to achieve complete hydrolysis.

#### 2.14.2. Gastrointestinal Digestion

Simulated gastric fluid (SGF) and simulated intestinal fluid (SIF) stock solutions were prepared. The synthetic peptide (20 mg) was dissolved in 5 mL phosphate buffer (pH 3.0), followed by addition of 4 mL of preheated SGF digestion solution (pH 3.0) and 1 mL of pepsin solution (2000 U/mL). The mixture was incubated and shaken (37 °C, 2 h, 300 rpm) to simulate the gastric phase of digestion. Subsequently, 8 mL of SIF digestion solution (pH 7.0) and 2 mL of trypsin and chymotrypsin mixture (concentrations of trypsin at 100 U/mL and chymotrypsin at 25 U/mL) were added to 10 mL of gastric digestion solution. The mixture was incubated at 37 °C with shaking for 3 h to simulate the intestinal phase of digestion. The digestion solution was further heated in boiling water for 15 min to terminate the digestion process.

#### 2.14.3. The Half Maximal Inhibitory Concentration

The *IC*_50_ of samples before and after digestion was determined, following the method described in Section 2.7. The experiment was designed with the following groups: the experimental groups included SP_FPL_ and SP_FPHFDL_ (synthetic peptides before digestion), and SP-SGD_FPL_ and SP-SGD_FPHFDL_ (after simulated gastrointestinal digestion with enzymes); the control group consisted of SP-SC_FPL_ and SP-SC_FPHFDL_ (peptides subjected to digestion without enzymes); and blank group (digestion fluid alone). The sample was diluted to 4 mM, 2 mM, 1 mM, 500 µM, and 250 µM using 100 mM Tris-HCl buffer (pH 8.0). The reaction activity was measured by mixing DPP-IV (2 µg/mL, 40 µL), peptide solutions (80 µL) and the substrate. The *IC*_50_ was calculated by fitting a curve with peptide concentration as the abscissa and DPP-IV activity as the ordinate.

### 2.15. Statistical Analysis

All experiments were repeated three times independently, and the results are presented as Mean ± standard deviation. IBM SPSS Statistics 29 software was used for statistical analysis to compare differences between groups using one-way ANOVA, followed by LSD post hoc tests. *p* < 0.05 was considered statistically significant. The results were plotted using SigmaPlot 15.0 software. One-way analysis of variance was used to compare the differences between groups, and LSD method was used for multiple comparisons. Statistical results were plotted using SigmaPlot15.0 software.

## 3. Results and Discussion

### 3.1. Bioactive Potential of Goat Blood Peptide Based on In Silico Analysis and Amino Acid Composition

In order to predict the bioactive potential of peptides derived from goat blood proteins and to rapidly screen for optimal enzymatic hydrolysis conditions, an *in silico* approach was employed to analyze five major proteins in goat blood: hemoglobin subunit alpha1, hemoglobin subunit alpha2, hemoglobin subunit beta-A, hemoglobin subunit beta-C, and albumin (Table S1). As shown in Figure S1, goat blood proteins exhibit significant potential as DPP-IV inhibitory peptide precursors, with *A* values of 0.6972, 0.6831, 0.6207, 0.6056, and 0.7111, which surpass those of sheep plasma protein (*A* = 0.6468), porcine plasma protein (*A* = 0.6438), and bovine plasma protein (*A* = 0.6509).

The amino acid composition of substrate is a crucial factor influencing bioactivity. The protein content was 88.2 g/100 g, with a total of 17 amino acids detected. The most abundant amino acids were Leu (12.51%), Ala (11.34%), Asp (10.25%), Val (9.26%), Lys (7.45%), Gly (7.42%), Glu (7.34%), Pro (6.59%), Ser (5.75%), and Phe (5.52%). Pork blood meal and beef blood meal exhibited similar amino acid compositions [33]. Previous studies indicated that the binding site of DPP-IV contained a hydrophobic pocket, and a typical characteristic of DPP-IV inhibitory peptides had hydrophobic amino acid residues at both terminals of peptide chain [34]. The proportion of hydrophobic amino acids in goat blood protein (56.10%) was significantly higher than that in porcine blood meal (44.54%) and bovine blood meal (45.07%), potentially contributing to its higher *A* value.

**Figure 1:**
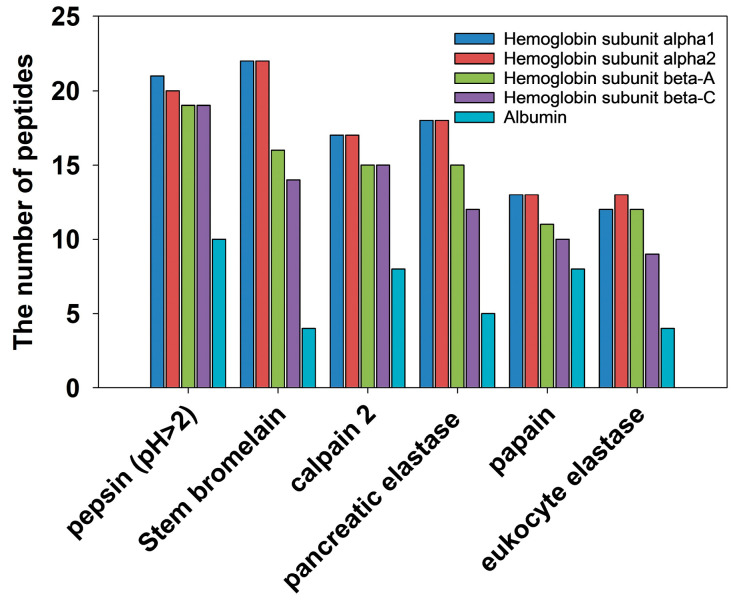
The predicted number of DPP-IV inhibitory peptides released from goat blood proteins via *in silico* hydrolysis.

*In silico* hydrolysis was performed to screen for the optimal enzymes. Results, illustrated in Figure 1, are ranked from highest to lowest according to the number of predicted DPP-IV inhibitory peptides released. Pepsin and bromelain yielded the highest numbers, with a total of 89 and a total of 78 peptides, respectively, surpassing calpain 2 (a total of 72) and pancreatic elastase (a total of 68).

Pepsin, characterized by broad cleavage specificity, demonstrates a high likelihood of cleaving peptide bonds at the C-terminal of Phe and Leu and the N-terminal of Ile, with some activity observed at other positions [35]. The highest content of Leu (12.51%) in goat blood protein suggests numerous potential cleavage sites, which may explain the superior hydrolysis performance predicted for pepsin in the *in silico* analysis. It is noteworthy that while pepsin exhibits optimal activity under acidic conditions (pH 2), the heme groups in goat blood protein are prone to precipitation within this pH range, potentially reducing substrate availability [36]. Furthermore, preliminary experiments indicated that pepsin demonstrated low hydrolysis efficiency (DH < 5%), and the resulting hydrolysates exhibited low DPP-IV inhibitory activity. This finding suggested that substrate accessibility is a critical factor limiting hydrolysis efficiency [37].

Bromelain exhibits a preference for cleaving peptide bonds between hydrophobic and basic amino acids [38]. This phenomenon aligns with the amino acid composition of goat blood proteins, characterized by a 65.02% proportion of hydrophobic and basic amino acids, leading to a greater diversity and quantity of generated peptides. Additionally, the cleavage specificity of bromelain promotes the enrichment of residues such as Ala and Leu. This is consistent with the structural characteristics of DPP-IV inhibitory peptides, which typically contain Ala, Gly, Leu, Val, or Phe at their termini [39]. Previous studies have shown that bromelain can effectively release DPP-IV inhibitory peptides through enzymatic hydrolysis of silkworm pupae protein, with the highest inhibitory activity (73.30%) at 8 h [16]. Moreover, Kamal [40] confirmed the advantage of bromelain in releasing DPP-IV inhibitory peptides, with 4 h hydrolysate demonstrating the optimal DPP-IV inhibition (*IC*_50_ = 1.22 ± 0.08 mg/mL). Building upon the screening results from *in silico* analysis and the theoretical advantages of bromelain, this study further validated the actual enzymatic efficiency and product activity of bromelain through experimental analysis.

### 3.2. Determination of DH and Molecular Weight (MW) Distribution

**Figure 2:**
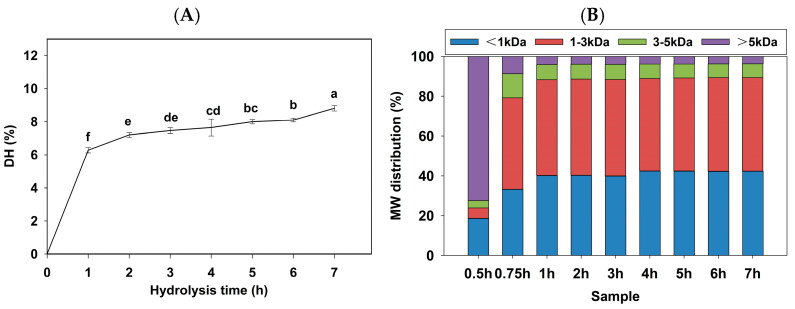
Degree of hydrolysis curve (**A**) and molecular weight distribution (**B**) of protein hydrolysate from goat blood protein. Different letters within the same group indicate statistically significant differences.

Protein hydrolysate from goat blood was prepared using bromelain, and the extent of peptide bond cleavage was assessed by DH (Figure 2A). The MW distribution of hydrolysates was analyzed using size exclusion chromatography (Figure 2B). A rapid degradation of goat blood protein was observed within the first hour, evidenced by a sharp decrease in the proportion of high molecular weight peptides (>5 kDa) from 72.31% to 4.07%. Meanwhile, the proportion of small molecular weight peptides (<1 kDa and 1–3 kDa) increased from 18.66% to 40.22% and from 5.21% to 48.07%, respectively, with a corresponding increase in DH to 6.28%. During the initial phase, bromelain showed the maximal enzymatic hydrolysis rate at high substrate concentrations [41]. The DH gradually increased to 8.81% from 1–7 h, attributed to substrate depletion and product inhibition. The MW distribution analysis revealed a slight decrease in the peptide fraction (1–3 kDa) from 48.07% to 46.97%, while peptide fraction (<1 kDa) increased from 40.22% to 42.38%, suggesting the formation of small bioactive fragments. A similar phenomenon was reported in the hydrolysis of apple seeds, where the hydrolysis rate decreased with increasing hydrolysis time, reaching a maximal DH of 14% at 90 min [42]. The dominant role of small peptides in bioactivity has been validated in other protein systems. For instance, quinoa hydrolysates obtained after 2 h pepsin–trypsin hydrolysis (DH = 27.27%) showed significantly higher bioactivity, which has a higher content of the <1 kDa fraction [43]. Furthermore, research on corn germ hydrolysis demonstrated that the <3 kDa fraction obtained by ultrafiltration exhibited significantly higher inhibitory effects than the unfractionated components [44]. Despite the relatively low DH observed in this study, the peptide fraction (<3 kDa) accounted for 89.35% of the hydrolysate at 7 h, indicating the effective release of small bioactive peptides. This correlation was further validated in subsequent DPP-IV inhibitory activity assays.

### 3.3. DPP-IV Inhibitory Activity of Protein Hydrolysates from Goat Blood

**Figure 3:**
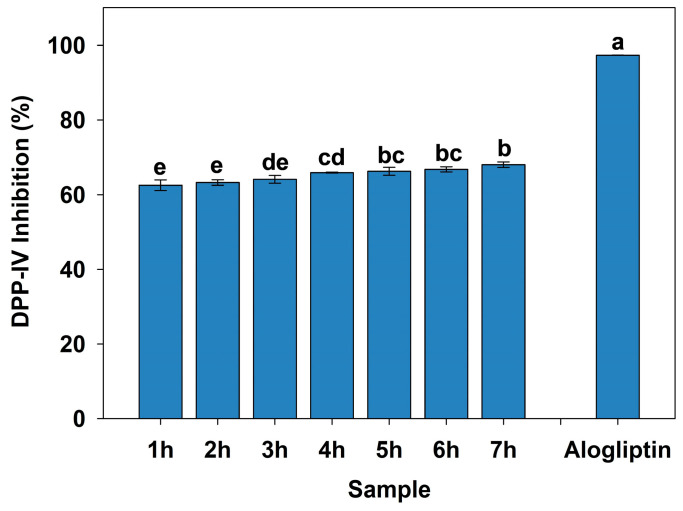
The DPP-IV inhibitory activities of protein hydrolysates at different hydrolysis times and Alogliptin. Different letters within the same group indicate statistically significant differences.

The *in vitro* DPP-IV inhibitory activity of hydrolysates from goat blood was evaluated, and the results are shown in Figure 3. The DPP-IV inhibitory activity of the positive control, alogliptin, was determined to be 97.35%. The DPP-IV inhibitory activity of the hydrolysate increased with increasing DH and the presence of small peptides, reaching a maximum at 7 h, with DPP-IV inhibitory activity of 68.00%. The 5 h hydrolysate exhibited a DPP-IV inhibitory rate of 66.23%, which was not significantly different from 7 h (*p* < 0.05). This observation may be attributed to substrate depletion or product inhibition effects, as also reported in the enzymatic hydrolysis of silkworm pupae protein [16]. Consequently, considering production time and practical applications in the food industry, the 5 h hydrolysate was selected for subsequent analysis.

After enzymatic hydrolysis with bromelain, several DPP-IV inhibitory peptides were released from goat blood proteins. The degree of hydrolysis, low molecular weight peptide content and DPP-IV inhibitory activity of hydrolysate were positively correlated. This phenomenon is consistent with previous findings, where the DPP-IV inhibitory activity of walnut protein hydrolysates increased with both DH and small peptide content; the 5 h hydrolysate exhibited the highest inhibitory activity of 33.90% (*IC*_50_ = 0.9 mg/mL) [45]. Most of the reported potent DPP-IV inhibitors (*IC*_50_ < 100 µM) are short peptides with 2–8 amino acid residues. Short peptides can more readily enter the DPP-IV active site and exert an inhibitory effect as competitive inhibitors [46,47]. The peptide (<1 kDa) fraction of goat skin collagen hydrolysates exhibited the highest proportion and the greatest DPP-IV inhibitory activity [48]. Therefore, hydrolysis of 1–3 kDa and the increased content of peptide (<1 kDa) are crucial factors, contributing to the enhanced DPP-IV inhibitory activity of 5 h hydrolysate.

### 3.4. Model Construction and Activity Prediction Based on Machine Learning

**Figure 4:**
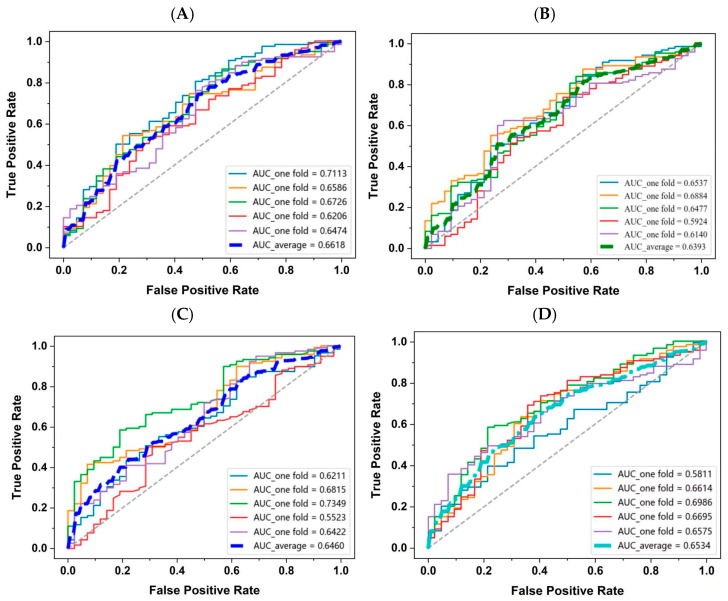

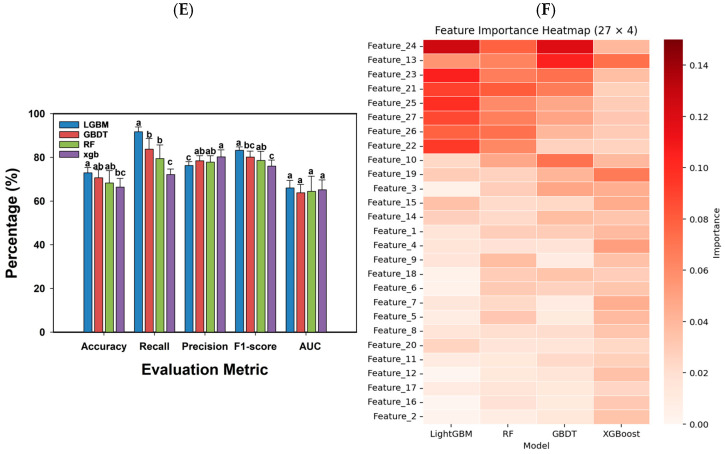
Evaluation indices of four machine learning models. ROC curves and AUC values of LightGBM (**A**), GBDT (**B**), RF (**C**) and XGBoost (**D**). Feature importance rankings (**F**) of LightGBM, GBDT, RF and XGBoost. The results (**E**) show the average value of each index, including accuracy, recall, precision, F1-score and AUC. Different letters within the same group indicate statistically significant differences.

To identify the optimal model for predicting DPP-IV inhibitory peptides, four different machine learning models were trained and validated. Five-fold cross-validation strategy was used to evaluate the generalization capability and stability of the model. As shown in Figure 4A–D, all ROC curves of the model were consistently and significantly above the random guessing line (diagonal). These indicate that the constructed model can stably distinguish DPP-IV inhibitory peptides from non-inhibitory peptides, demonstrating fundamental predictive ability. However, the fluctuation in AUC values also suggests that the model’s performance exhibits some sensitivity to the specific partitioning of the training data. The model’s discriminatory power under the current feature and algorithmic framework still requires further improvement. This could be attributed to the inherent complexity of the peptide sequence–activity relationship, or additionally, to the fact that the negative dataset consisted of reported low-activity inhibitory peptides rather than random peptides, making the classification task more challenging.

As shown in Figure 4E,F, the LightGBM mode showed better overall performance, with the recall proportion of 91.84% and an F1-score of 83.39%, both significantly higher than other models (*p* < 0.05). This data indicated enhanced sensitivity and balanced classification capability for identifying active peptides. The high recall suggested effective capture of true positives, critical for minimizing false negatives in virtual screening. Feature importance analysis revealed that Feature_24 (average molecular weight) consistently ranked highly across all models, particularly in LightGBM and GBDT, suggesting that DPP-IV inhibitory peptides are associated with a higher average molecular weight.

The GBDT model exhibited balanced performance metrics, with all indicators at moderate to high levels, notably outperforming XGBoost in Recall (83.87%). Its high weight assigned to Feature_24 (0.1192), consistent with LightGB, indicates similar importance judgments for this critical feature among gradient boosting algorithms. The RF model showed stable precision (77.96%) but relatively lower recall (79.58%), suggesting an advantage in reducing false positives while potentially missing some active peptides. The feature importance distribution indicated a strong reliance on Feature_21 (hydrophobicity first-order autocorrelation). XGBoost performed comparatively weaker in this study, despite achieving the highest precision (80.37%), with the lowest another indicators, indicating a higher risk of false negatives. This may be attributable to suboptimal parameter tuning or feature interaction handling. Its feature importance analysis highlighted a dependence on Feature_13 (relative frequency of Pro occurrence), suggesting a distinct learning mechanism from other models.

A comprehensive analysis of feature importance rankings across the four models revealed that Feature_24 (Mass) and Feature_13 (Pro frequency) consistently appeared among the top ten features, underscoring their universal relevance in predicting DPP-IV inhibitory activity. Additionally, features such as Feature_23 (hydrophilicity), Feature_21 (hydrophobicity autocorrelation), Feature_25 (pK_a1_), Feature_27 (pI), Feature_10 (Lue frequency), Feature_19 (Trp frequency), and Feature_26 (pK_a2_) ranked within the top ten in three models. Notably, the high importance of hydrophobic autocorrelation (Feature_21) and hydrophilicity (Feature_23) suggests that the hydrophobic interactions are determinants of peptides binding to DPP-IV, which is consistent with the distribution of hydrophobic cavities in DPP-IV active sites [49]. Peptides with higher hydrophobicity are more likely to access these pockets, acting as competitive inhibitors [50].

Furthermore, the residues such as Pro, Leu, and Trp (corresponding to Features_13, 10, and 19) were identified as highly significant across multiple models, aligning with the reported literature. It has been shown that a key feature of potent DPP-IV inhibitors is Pro positioned at the second N-terminal residue, which correlates with the high frequency of Pro in inhibitory peptides [51]. Pro enhances hydrophobicity and promotes β-turn formation, driven by the geometric constraints of its rigid pyrrolidine ring [52]. This structural feature exposes cleavage sites for DPP-IV and facilitates molecular recognition and hydrophobic interactions at the catalytic center. The aliphatic amino acid Leu can also form stable hydrophobic interactions in the S1 binding pocket of DPP-IV. Trp, with its planar indole side chain (a phenyl ring fused to a pyrrole ring), exhibits strong hydrophobicity and aromaticity. Its significance lies not only in its hydrophobic nature, favoring binding to hydrophobic pockets, but also in its capacity to participate in π-π stacking interactions with aromatic residues (Tyr547, Tyr666, and Tyr662) within the catalytic center [5], which are crucial for stabilizing peptide–enzyme complexes. Overall, compared to the other models, LightGBM demonstrated superior overall performance and was selected as the optimal model for DPP-IV inhibitory peptide screening, while the prediction values of LightGBM model in machine learning were listed in Table 1.

### 3.5. Peptide Identification by Peptidomics

For the purpose of exploring peptide sequences with high DPP-IV inhibitory activity, LC-MS/MS analysis was performed for the 5 h protein hydrolysate, leading to the identification of 37,199 peptides that consist of 2–27 amino acids and correspond to the m/z range of 340–1100. The following screening criteria were applied: (1) relative abundance > 1 × 10^7^; (2) peptide chain length < 10 amino acids; (3) PeptideRanker score > 0.9. A total of 529 candidate peptides were selected. Further screening was performed based on the fact that DPP-IV preferentially cleaves peptides with Pro or Ala at the second position from the N-terminus. Nine potent DPP-IV inhibitory peptides derived from goat blood protein were identified, as listed in Table 1. Peptides derived from hemoglobin subunit alpha included FPH, FPHF, FPHFD, and FPHFDL, while YPWTQRFF and YPW originated from hemoglobin subunit beta. HPYF was derived from albumin and FPFA originated from alpha-2-macroglobulin isoform X1/2. FPL originated from alpha-2-macroglobulin isoform X1. Among the tripeptides screened, FPL (Phe-Pro-Leu) exhibited the highest PeptideRanker score (0.9790), LightGBM model predicted (0.9863), and its hydrophobic N-terminal Phe may facilitate binding to the DPP-IV catalytic domain. Previous studies have indicated that the presence of hydrophobic amino acids (e.g., Gly, Ala, Pro, Ile, Lue, Phe) at the N-terminal of low molecular weight peptides is associated with higher DPP-IV inhibitory properties, as these residues can interact with the hydrophobic active site of the DPP-IV pocket, leading to tight binding [53]. Among the peptides comprising more than four amino acids, FPHFDL had a relative abundance of 4.33 × 10^9^, significantly higher than the homologous sequences FPHF (2.56 × 10^9^) and FPHFD (8.32 × 10^8^). Both the N- and C-terminal of such peptides were hydrophobic, which is consistent with the characteristics of DPP-IV inhibitors. Meanwhile, the predicted value of LightGBM model for FPHFDL reached 0.9782. Additionally, it has been suggested that highly effective DPP-IV peptides with ≥4 amino acids often have Pro, Leu or Arg at the C-terminal [46]. Therefore, FPL and FPHFDL were selected for further verification of DPP-IV inhibitory activity.

### 3.6. Inhibition Kinetics of Synthetic Peptides

**Figure 5:**
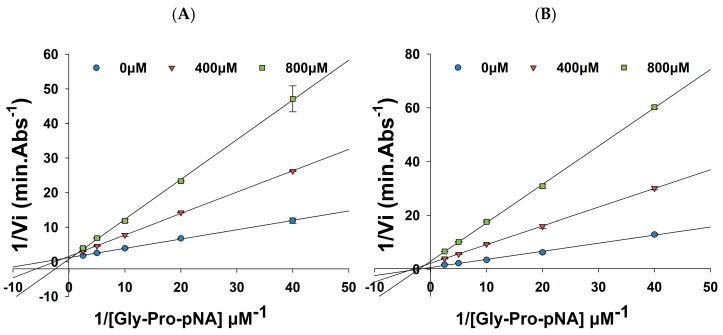
Lineweaver–Burk plots of DPP-IV inhibition by FPL (**A**) and FPHFDL (**B**) at different concentrations.

The Lineweaver–Burk plot in Figure 5 presents the kinetic data for DPP-IV hydrolysis of the substrate Gly-Pro-pNA in the presence of varying concentrations of the inhibitory peptide (FPL or FPHFDL), used to determine the inhibition type. Upon addition of FPL (Figure 5A), the *K_m_* value significantly increased from 0.2684 µM to 0.4196 µM (*p* < 0.05), while *V_max_* did not change significantly (*p* > 0.05), indicating that FPL does not affect the maximal reaction rate of the enzyme, suggesting that FPL acts as a competitive inhibitor of DPP-IV. Its structure is similar to the natural substrate of DPP-IV, allowing it to competitively bind to the active site of enzyme, thereby limiting the binding and catalytic action of DPP-IV on the substrate [54]. DPP-IV can recognize and cleave Pro or Ala at the second position from the N-terminal. For instance, the *IC*_50_ values of Diprotin A (IPI) and Diprotin B (VPL) were 3.5 µM and 15.8 µM, respectively; both are known to be strong competitive inhibitors of DPP-IV [55]. The inhibitory mechanism of FPL is consistent with that of IPI, and both peptides contain hydrophobic amino acids, suggesting that N-terminal hydrophobic residues (Trp, Ile, Phe, and Leu) often exhibit strong DPP-IV inhibitory activity due to their interaction with the hydrophobic cavity of the DPP-IV active site. Such hydrophobic interactions can enhance binding stability [46].

The initial velocity changes at different substrate concentrations in the presence of 0 µM, 400 µM, and 800 µM FPHFDL are presented in Figure 5B. After adding 400 µM FPHFDL, the *V_max_* of DPP-IV significantly decreased (*p* < 0.05) from 1.4428 (ΔA/h) to 0.4580 (ΔA/h), while *K_m_* did not change significantly (*p* > 0.05). The lines intersect on the negative X-axis, indicating that FPHFDL is a non-competitive inhibitor, and the inhibition of DPP-IV is independent of substrate concentration. Therefore, it can be inferred that the inhibitor binds to an allosteric site outside the enzyme’s active center, leading to a conformational change in the enzyme that reduces the catalytic rate and substrate binding. Studies on anthraquinones have revealed that alizarin red S (*IC*_50_ = 8.33 µM) and emodin (*IC*_50_ = 3.81 µM) can act as non-competitive inhibitors of DPP-IV, producing a high degree of inhibition [56]. The peptides LDKVFR and VLATSGPG, identified from salmon skin collagen hydrolysate, also exhibit non-competitive inhibition [57]. Currently, a study on DPP-IV inhibitory peptides primarily focuses on competitive inhibition, with fewer reports on non-competitive inhibitors. However, application of competitive inhibitory peptides faces challenges such as degradation and loss of inhibitory activity. Additionally, reliance on competitive inhibitors may neglect the non-enzymatic functions of DPP-IV, such as inflammation regulation and cell signal transduction (e.g., CD26) [58]. Different types of inhibition can also contribute to the exploration of new biological functions of DPP-IV, providing novel specific targets for disease treatment [56]. The *K_i_* values for the two peptides were calculated, revealing *K_i_* = 186 µM for FPHFDL, which is less than *K_i_* = 709 µM for FPL, indicating that FPHFDL interacts more closely with DPP-IV as a non-competitive inhibition. Non-competitive inhibitors generally exhibit lower *K_i_* values.

### 3.7. Molecular Docking

Molecular docking and molecular dynamics were used to predict the binding modes of goat blood peptides (FPL and FPHFDL) with DPP-IV and explore their inhibition mechanisms. It was observed that hydrogen bond binding sites and residues generating hydrophobic interactions at the binding end (90 ns and 100 ns) did not differ significantly (Figure S2), indicating that they were stable structures. In the blind docking results, the binding energy of FPHFDL (−9.2 kcal/mol) was lower than that of FPL (−7.3 kcal/mol). Similarly, following molecular dynamics simulation, the binding energy of FPHFDL (−33.43 kcal/mol) remained lower than that of FPL (−16.51 kcal/mol). This computational evidence aligns with experimental kinetics: the lower *K_i_* value measured for FPHFDL confirms its enhanced binding to DPP-IV.

However, this is contrary to the observed *IC*_50_ values, where FPL (321.5 ± 11.3 µM) exhibited a lower *IC*_50_ than FPHFDL (465.6 ± 11.6 µM), suggesting that FPL has a stronger inhibitory effect on DPP-IV. This contradiction can be explained by the difference in inhibition mode. FPHFDL acts as a mixed-competitive inhibitor, a portion of which may bind to the allosteric site of DPP-IV. Although the binding free energy is high, the inhibitory efficacy is suboptimal because it indirectly affects catalytic efficiency through conformational changes. In contrast, FPL exhibits competitive inhibition, despite its weaker binding free energy, it effectively blocks substrate binding by directly occupying the active site of DPP-IV.

During blind docking, FPL formed three hydrogen bonds with DPP-IV. FPL interacts with both the S1 pocket (Tyr547, with two hydrogen bonds) and S2 subsite (Arg125, with one hydrogen bonds).

**Figure 6:**
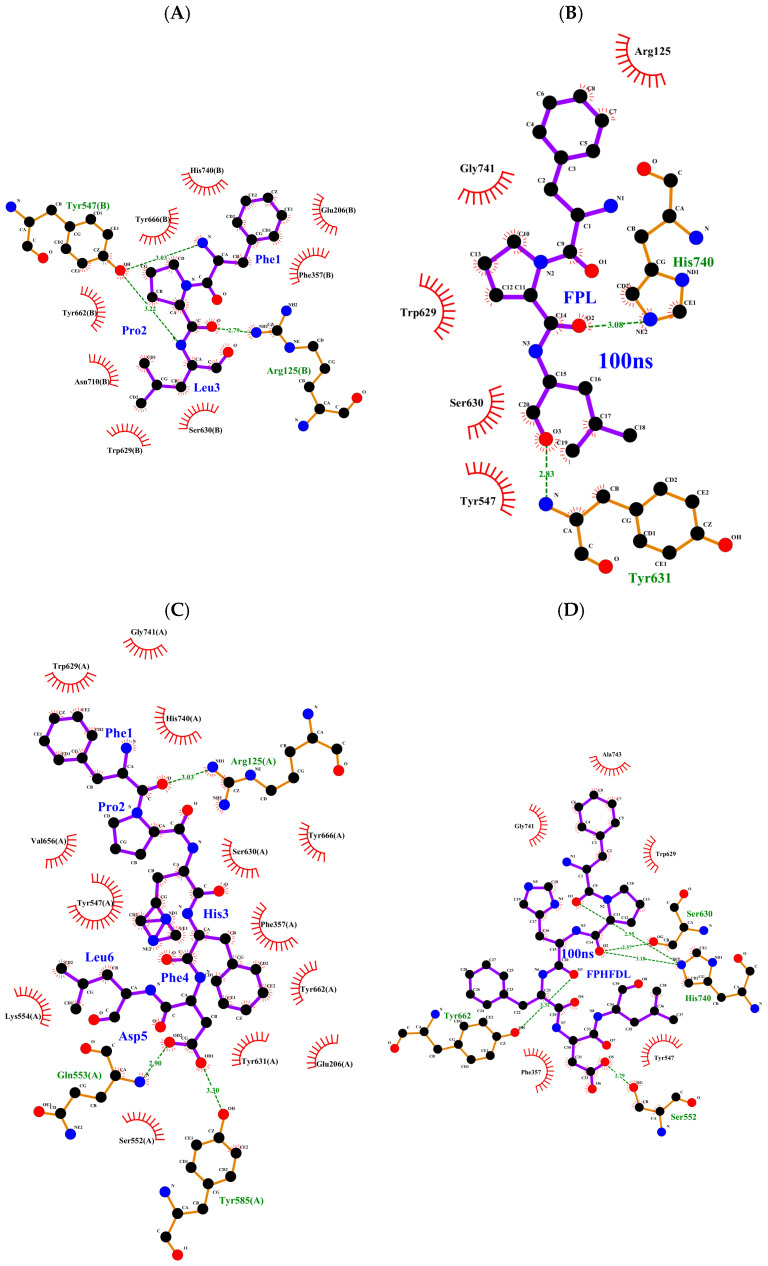
Molecular docking analysis of DPP-IV (PDB ID: 1WCY) with synthetic peptides FPL and FPHFDL. (**A**) Ligand interaction diagram of FPL-DPP-IV complex during molecular docking. (**B**) Ligand interaction diagram of FPL-DPP-IV complex during molecular dynamics simulation. (**C**) Ligand interaction diagram of FPHFDL-DPP-IV complex during molecular docking. (**D**) Ligand interaction diagram of FPHFDL-DPP-IV complex during molecular docking. Note: Hydrogen bonds are indicated by green dashed lines with distance values. Hydrophobic interactions are represented by red spoke arcs (non-ligand residues).

FPL displayed hydrophobic interactions with several residues (Table 2), with significantly greater contributions originating from the S1 pocket (Tyr666, Tyr662, Asn710, His740, Ser630) compared to those from the S2 subsite (Glu206, Phe357) (Figure 6A). After MD simulation, FPL formed two hydrogen bonds with His740 and Tyr631 (S1 pocket). Additional hydrophobic interactions occurred with Arg125, Gly741, Trp629, Ser630, and Tyr547, mainly localized in the S1 pocket (Figure 6B). His740 (S1 pocket) is a component of DPP-IV catalytic triad, responsible for dipeptide cleavage. Tyr547 and Tyr631, also within S1 pocket, play a role in stabilizing the catalytic transition state conformation by the oxyanion hole [57]. Arg125 (S2 subsite) helps maintain the conformational stability of the FPL-DPP-IV complex [49]. Although the specific residues involved in binding slightly varied following molecular dynamics simulations, they are predominantly located in the S1 pocket.

The competitive inhibition mechanism of FPL arises from its occupancy of the S1 pocket situated within the C-terminal α/β hydrolase domain (Gln508-Pro766, along with segment Leu45-Val55). This pocket serves two critical functions: (i) providing a hydrophobic microenvironment for ligand, and (ii) recognizing the N-terminal P1 residue (Pro or Ala), which is essential for substrate engagement and dipeptide bond cleavage. This contributes to the creation of highly specific competitive binding sites [59]. A similar binding pattern was observed in the crystal structure of the PkDPP-IV + ILAPPER complex, where Leu2 non-covalently interacted with Ser630 in the catalytic triad by two hydrogen bonds, and ILAPPER also acted as a reversible competitive inhibitor [54].

After molecular dynamics simulations, FPHFDL interacted with DPP-IV through five hydrogen bonds (Figure 6C). The N-terminal residues Phe1 and Pro2 can bind to the catalytic triad of S1 pocket (His740, Ser630) [60]. His3 formed a hydrogen bond with Tyr662 (S1 pocket), while the carboxyl terminus of the Asp5 R-group established a hydrogen bond with Ser552, whose mechanism is not yet fully understood (Figure 6D). Hydrophobic interactions were observed with Gly741, Trp629, Tyr547, Phe357, and Ala743. FPHFDL primarily binds to the S1 pocket and Ser552 (outside the active center). Enzyme kinetic analysis indicated that FPHFDL acted as a non-competitive inhibitor, and its non-competitive inhibition characteristics may be linked to the binding of Ser552. Energy decomposition analysis suggested that Ser552 makes a significant contribution to the binding energy.

### 3.8. Molecular Dynamics Simulation

**Figure 7:**
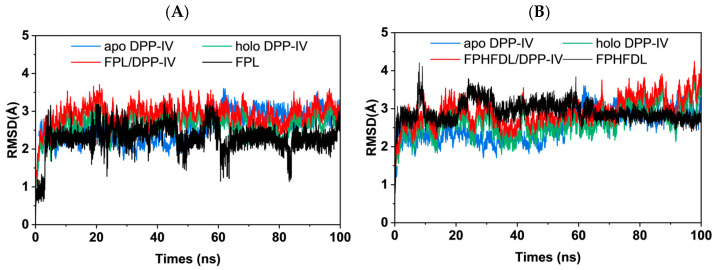

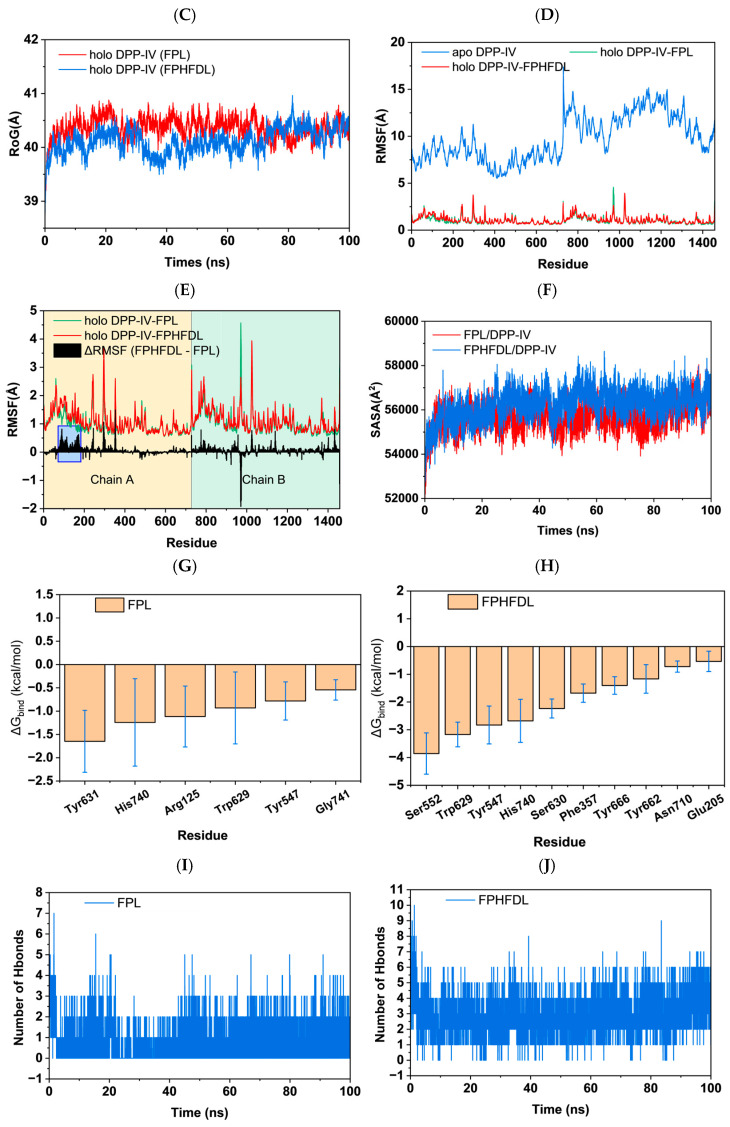
Molecular dynamics simulation results of apo DPP-IV and synthetic peptides FPL and FPHFDL. (**A**) RMSD of apo DPP-IV and FPL/DPP-IV; (**B**) RMSD of apo DPP-IV and FPHFDL/DPP-IV; (**C**) RMSF of FPL/DPP-IV and FPHFDL/DPP-IV; (**D**) RMSF of DPP-IV with FPL or FPHFDL and ΔRMSF (FPHFDL-FPL); (**E**) RoG of FPL/DPP-IV and FPHFDL/DPP-IV; (**F**) SASA of FPL/DPP-IV and FPHFDL/DPP-IV; (**G**) Binding energy contributions from DPP-IV residues in FPL/DPP-IV; (**H**) Binding energy contributions from DPP-IV residues in FPHFDL/DPP-IV; (**I**) Hydrogen bond number between FPL and DPP-IV; (**J**) Hydrogen bond number between FPHFDL and DPP-IV.

Based on the molecular docking results, all-atom molecular dynamics simulations were conducted to investigate the movement and stability of the ligand within the binding site. RMSD and RMSF were employed to characterize the conformational stability and local flexibility of the complexes, respectively [61]. RMSD reflects the overall structural deviation of the protein relative to the initial conformation, with its standard deviation quantifying the range of dynamic fluctuations, while RMSF indicates the local flexibility changes of specific residues during the simulation [62]. As shown in Figure 7A,B, all systems remained stable without significant fluctuations or conformational collapse during the simulation (holo DPP-IVFPL RMSD = 2.631 ± 0.295 Å, holo DPP-IVFPHFDL RMSD = 2.648 ± 0.383 Å), which provides the necessary structural basis for the binding of ligand.

Comparative analysis of the binding characteristics of the two peptides revealed that the FPL exhibited a lower average RMSD and a higher standard deviation (2.300 ± 0.372 Å), indicating less overall displacement in the active site but significant local conformational change. Conversely, FPHFDL displayed a higher average RMSD with a lower standard deviation (2.924 ± 0.262 Å), suggesting a greater overall deviation after simulations, accompanied by relative stability in pose. This difference may reflect distinct inhibitory mechanisms. FPL, as a competitive inhibitor, dynamically adapts in the active site, whereas FPHFDL, as a non-competitive inhibitor, binds in allosteric sites directly to regulate activity. Supporting this inference, the RMSD standard deviation of DPP-IV in the FPHFDL system (0.385 Å) was greater than that in the FPL system (0.295 Å), indicating that allosteric effects may increase the fluctuation of the entire receptor.

The conclusions can be further verified by RMSF analysis. As shown in Figure 7C, the RMSF values of apo DPP-IV were all higher than 5 Å. After peptide binding, the RMSF values of most sequence segments decreased to below 2 Å, suggesting that the ligand binding enhanced receptor rigidity. Notably, the average RMSF of the receptor in the FPHFDL system (1.078 Å) was higher, indicating that the DPP-IV in the FPHFDL system exhibited greater flexibility during binding, possibly undergoing conformational changes. Further analysis of residues where conformational changes may occur in DPP-IV upon FPHFDL binding is presented in Figure 7D. Since FPHFDL binds to chain A of DPP-IV and FPL binds to chain B of DPP-IV, the RMSF values of the residues 118–162 and 162–216 regions fluctuated after binding FPHFDL, particularly near the S2 active site (Arg125, Glu205, Glu206, Val207, Ser209), indicating that these residues underwent some conformational changes after binding.

RoG and SASA analyses were performed to assess the stability [63]. RoG reflects the compactness of the system, while SASA indicates the area of the complex accessible to the aqueous solvent. Figure 7E,F illustrate the time-dependent changes in RoG and SASA for DPP-IV during the molecular dynamics simulations, suggesting that DPP-IV maintained a stable overall conformation in both systems, as evidenced by the reasonable fluctuations in RoG and SASA values.

Binding free energy calculations and residue decomposition were performed using the MM-GBSA method for the late stage (90–100 ns) of the molecular dynamics simulation. The total binding free energy (Δ*G*_bind_ = −33.43 ± 3.69 kcal/mol) for the FPHFDL/DPP-IV system was significantly superior to that of the FPL/DPP-IV system (Δ*G*_bind_ = −16.51 ± 3.31 kcal/mol), indicating a stronger binding affinity and more stable conformation of FPHFDL to DPP-IV. Energy decomposition analysis revealed that van der Waals interactions (Δ*E*_vdW_) and nonpolar solvation energy (Δ*G*_SA_) were the primary driving forces for the binding of both peptides to DPP-IV, with van der Waals forces playing a dominant role. Specifically, the van der Waals interaction energy of FPHFDL (Δ*E*_vdW_ = −53.35 ± 3.43 kcal/mol) was significantly stronger than that of FPL (Δ*E*_vdW_ = −29.09 ± 3.43 kcal/mol). This difference is closely related to the longer peptide chain structure of FPHFDL, which allows for complementary interactions with the hydrophobic pocket through additional hydrophobic residues (Phe), significantly enhancing binding stability. Subsequently, the MM-GBSA energy decomposition technique was employed to identify amino acids contributing more than 0.5 kcal/mol to the binding of FPL/DPP-IV and FPHFDL/DPP-IV (Figure 7G,H). For the FPL/DPP-IV complex, key binding residues included Tyr631, His740, Arg125, Trp629, Tyr547, and Gly741, primarily located in the S1 pocket of DPP-IV, with higher error indicating possible instability in binding. For FPHFDL/DPP-IV complex, key residues included Ser552, Trp629, Tyr547, His740, Ser630, Phe357, Tyr666, Tyr662, and Asn710. Among these, Tyr547, His740, Ser630, Tyr666, Tyr662, and Asn710 are situated at the S1 active site of DPP-IV. Glu205 and Phe357 belong to the S2 binding site. Notably, the roles of Ser552 and Trp629 have not been reported, but they are crucial for FPHFDL binding and may relate to its non-competitive inhibitory characteristics. LigPlot+ visualization results also observed that Ser552 enhances the binding of the FPHFDL to DPP-IV through hydrogen bonding, while Trp629 contributes to binding affinity through hydrophobic interactions.

Hydrogen bond, a key non-covalent interaction, was analyzed to explain the differences in binding modes. Hydrogen bond formation between the ligands (FPL and FPHFDL) and DPP-IV was monitored throughout the 0–100 ns molecular dynamics simulations (10,000 conformations). Figure 7I,J show marked differences in the binding of the two ligands. The FPHFDL/DPP-IV complex showed greater dynamic stability, with 53.4% of the total conformations exhibiting a high number of hydrogen bonds (≥3). Additionally, 74.8% conformations maintained three or more hydrogen bonds during the late simulation period (80–100 ns), indicating continuous and stable hydrogen bond interactions. Conversely, FPL displayed a significantly lower proportion of high hydrogen bond conformations (≥3), only 4.5%. In the later stages of the simulation (80–100 ns), the FPL/DPP-IV complex exhibited only one hydrogen bond (42.9%). This disparity may be attributed to the length and characteristics of the residues. FPHFDL, with its longer sequence, achieves stronger binding through the synergistic effects of hydrogen bonds and van der Waals forces. The presence of hydrophilic residues at positions three and five in the peptide chain may contribute to the altered inhibitory mode and tighter binding, consistent with the *K_i_* results. Longer peptides can provide more hydrogen bond acceptors or donors, and the presence of hydrophilic groups may generate electrostatic interactions with surrounding residues, thereby enhancing binding stability. Similar findings were reported, where the YYGYTGAFR exhibited lower binding free energy and *K_i_* values [57].

### 3.9. IC_50_ Value and Digestive Stability

**Figure 8:**
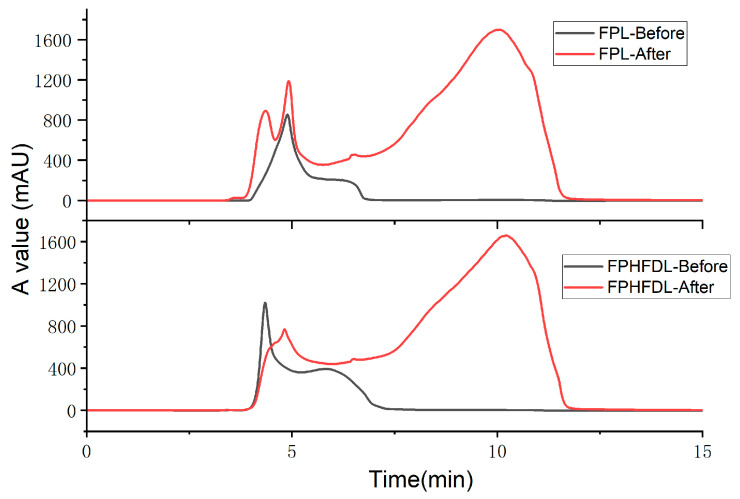
The HPLC chromatograms of FPL and PFHFDL before and after simulated gastrointestinal digestion.

*In vivo* the hypoglycemic effect of DPP-IV inhibitory peptides is closely associated with their gastrointestinal digestive stability. The DPP-IV inhibitory activity of peptide FPL was significantly altered after treatment. The IC_50_ of the SP_FPL_ was 321.5 ± 11.3 µM. In contrast, the IC_50_ values of both the digested sample (SP-SGD_FPL_, 425.5 ± 1.6 µM) and the enzyme-free control (SP-SC_FPL_, 426.0 ± 5.4 µM) were showed no significant difference from each other. This indicated that the loss of inhibitory activity was not caused by enzymatic cleavage during digestion, but rather by the acidic conditions of the simulated gastrointestinal process. This conclusion was further supported by SEC analysis, where the SP-SGD_FPL_ sample exhibited an earlier-eluting peak compared to the intact peptide, suggesting the formation of higher molecular weight aggregates under acidic conditions. Collectively, the acidic environment may impact aggregation of FPL, which consequently reduced its bioactivity (Figure 8).

For peptide FPHFDL, a contrasting trend was observed. The *IC*_50_ of the SP_FPHFDL_ was 465.6 ± 11.6 µM. After simulated digestion, the IC_50_ of SP-SGD_FPHFDL_ significantly decreased to 247.9 ± 2.7 µM, while the enzyme-free control (SP-SC_FPHFDL_) showed a value of 477.2 ± 13.2 µM, which was not significantly different from SP_FPHFDL_. The significant decrease in IC_50_ specifically in the digested sample demonstrated that the enhancement of DPP-IV inhibitory activity was due to enzymatic hydrolysis during the digestion process, rather than the acidic environment. This conclusion was corroborated by SEC analysis. The chromatogram of SP-SGD_FPHFDL_ showed that the main peak shifted to a longer retention time, which corresponds to a lower molecular weight. Additionally, a shoulder peak developed. This profile is consistent with the cleavage of the parent peptide by digestive enzymes (pepsin, trypsin, and chymotrypsin) into smaller peptide fragments. The “Enzymes-action” function of BIOPEP-UWM was utilized to predict the digestion results of FPL and FPHFDL.

Pepsin preferentially cleaves at hydrophobic amino acids, specifically at the N-terminus of Met, Glu, Leu, and Phe, as well as the C-terminus of Phe, Ile, Tyr, and Trp [35,64]. Suwareh et al. demonstrated that pepsin has the highest cleavage probability for the Phe-Asp (60%) and Asp-Leu (50%) [35]. Therefore, FPHFDL may be hydrolyzed into fragments such as FPHF, FPHFD, DL, and free amino acids (Asp and Leu). Trypsin can hydrolyze peptide bonds formed by the C-terminal of basic amino acids (His, Lys, Arg). Chymotrypsin can cleave peptide bonds formed by the C-terminus of aromatic amino acids (Phe, Tyr and Trp). Potential small peptides produced from these processes include FPH, FDL, FPHF, FPHFD, and DL, which may have higher DPP-IV inhibitory activity. Previous studies suggest that dipeptides (PH and DL) have weak inhibitory activity (<12%) [65]. Further investigations will be conducted to analyze the activity of these digested products. Although FPHFDL is not highly stable, its digestion products still retain significant hypoglycemic potential, warranting further exploration.

## 4. Conclusions

In this study, *in silico* analysis successfully predicted the potential of goat blood proteins as DPP-IV inhibitory peptides, identifying bromelain as the optimal protease. *In vitro* targeted enzymatic hydrolysis of goat blood protein using bromelain yielded a hydrolysate with DPP-IV inhibitory activity of 66.23% at 5 h. LightGBM was utilized to predict the DPP-IV inhibitory activity of peptides derived from goat blood protein. Nine novel peptides with potent DPP-IV inhibitory potential were identified (FPL, YPW, FPH, FPHF, FPHFD, FPHFDL, FPFA, HPYF, YPWTQRFF) using machine learning and virtual screening approaches. Notably, FPL exhibited a competitive inhibition mode, primarily binding to the S1 pocket of the DPP-IV through hydrogen bonds and hydrophobic interaction, demonstrating superior inhibitory efficiency compared to FPHFDL. Conversely, FPHFDL displayed non-competitive inhibition characteristics, illustrating a higher binding affinity through strong van der Waals interactions and hydrogen bonds involving key residues Ser552 and Trp629, although its *IC*_50_ value remained relatively higher. Notably, the *IC*_50_ value of FPHFDL decreased further after *in vitro* simulated gastrointestinal digestion, suggesting potential degradation into smaller, more active peptide fragments. However, the specific molecular mechanisms and *in vivo* hypoglycemic effects require further experimental validation.

## Figures and Tables

**Table 1:** LC-MS/MS results of high-confidence peptides identified from goat blood-derived protein hydrolysate.

Peptides	Peak Area	PeptideRanker	LightGBM	Protein Precursor
FPL	2.27 × 10^7^	0.9790	0.9863	Alpha-2-macroglobulin isoform X1 [*Capra hircus*]
YPW	3.12 × 10^7^	0.9751	0.9773	Hemoglobin subunit betaA/C [*Capra hircus cretica*]
FPH	1.02 × 10^9^	0.9401	0.9324	Chain A, Hemoglobin subunit alpha-1/2 [*Capra hircus*]
FPHF	2.56 × 10^9^	0.9872	0.7784	Chain A, Hemoglobin subunit alpha-1/2 [*Capra hircus*]
FPHFD	8.32 × 10^8^	0.9274	0.5945	Chain A, Hemoglobin subunit alpha-1/2 [*Capra hircus*]
FPHFDL	4.33 × 10^9^	0.9448	0.9782	Chain A, Hemoglobin subunit alpha-1/2 [*Capra hircus*]
FPFA	1.28 × 10^7^	0.9843	0.8665	Alpha-2-macroglobulin isoform X1/2 [*Capra hircus*]
HPYF	7.75 × 10^8^	0.9396	0.8794	Chain A, Albumin [*Capra hircus*]
YPWTQRFF	2.26 × 10^8^	0.9556	0.9425	Hemoglobin subunit betaA/C [*Capra hircus cretica*]

**Table 2:** Molecular docking data of DPP-IV (1WCY) and peptides (FPL and FPHFDL).

Docking Method	Peptides	Vina Score/Δ*G*_bind_ (kcal/mol)	Binding Sites with Hydrogen Bond		Interaction Sites by Hydrophobic Interaction
			Amino Acid Residue	Binding Sites (Length)	
Blind docking	FPL	−7.3	Phe1	Tyr547 (3.03 Å)	Glu206, Tyr666, Phe357, Tyr662, Asn710, His740, Ser630, Trp629.
			Pro2	Arg125 (2.79 Å)	
			Leu3	Tyr547 (3.22 Å)	
	FPHFDL	−9.2	Phe1	Arg125 (3.03 Å)	Glu206, Trp629, Gly741, His740, Tyr666, Val656, Tyr547, Ser630, Phe357, Tyr662, Lys554, Tyr631, Ser552.
			Asp5	Tyr585 (3.30 Å)	
			Asp5	Gln553 (2.90 Å)	
Molecular dynamics simulation (100 ns)	FPL	−16.51	Pro2	His740 (3.08 Å)	Gly741, Trp629, Tyr547, Arg125, Ser630
			Leu3	Tyr631 (2.83 Å)	
	FPHFDL	−33.43	Phe1	Hie740 (2.95 Å)	Gly741, Trp629, Tyr547, Phe357, Ala743
			Pro2	Ser630 (2.57 Å)	
			Pro2	Hie740 (3.18 Å)	
			His3	Tyr662 (3.32 Å)	
			Asp5	Ser552 (2.79 Å)	

## Data Availability

The original contributions presented in this study are included in the article/Supplementary materials. Further inquiries can be directed to the corresponding authors.

## Supplementary Materials

The following supporting information can be downloaded at: https://www.mdpi.com/article/10.3390/foods15020398/s1. Figure S1 The predicted A values of blood proteins in different species. Figure S2 Molecular docking analysis of DPP-IV enzyme (PDB ID: 1WCY) with synthetic peptides FPL and FPHFDL. Table S1 Characteristics of the main proteins in *Capra hircus* blood. Table S2 Characteristics of the enzymes used for *in silico* analysis.

## Abbreviations

DPP-IV, dipeptidyl peptidase IV; GLP-1, glucagonlike peptide 1; GIP, glucose-dependent insulinotropic polypeptide; DH, degree of hydrolysis; LightGBM, Light Gradient Boosting Machine; GBDT, Gradient Boosting Decision Tree; RF, Random Forest; XGBoost, Extreme Gradient Boosting; T2DM, Type 2 diabetes mellitus; MW, molecular weight.
