# Supplementary material for: AI-Driven Rapid Screening and Characterization of Dipeptidyl Peptidase-IV (DPP-IV) Inhibitory Peptides from Goat Blood Proteins: An Integrative In Silico and Experimental Strategy
Source: Foods. 2026 Jan 22;15(2):398. doi: 10.3390/foods15020398 (PMC12840617; doi:10.3390/foods15020398)
Supplement: Supplementary file 1 [file foods-15-00398-s001.zip › foods-4054420-supplementary.pdf]

Table S1 Characteristics of the main proteins in *Capra hircus* blood

Name	ID	Length (aa)	Mass (Da)	A value		Amino acid sequence
				DPP-IV inhibitor	ACE inhibitor	
Hemoglobin subunit alpha1	P0CH25	142	15164	0.6972	0.4789	MVLSAADKSNVKAAWGKVGGNAGAYGAEALERMFL<br>SFPTTKTYFPHFDLSHGSAQVKGHGEKVAAALTKAVG<br>HLDDLPGTLSDLSDLHAHKLRVDPVNFKLLSHSLLVTL<br>ACHLPNDFTPAVHASLDKFLANVSTVLTSKYR
Hemoglobin subunit alpha2	P0CH26	142	15191	0.6831	0.4718	MVLSAADKSNVKAAWGKVGSNAGAYGAEALERMFL<br>SFPTTKTYFPHFDLSHGSAQVKGHGEKVAAALTKAVG<br>HLDDLPGTLSDLSDLHAHKLRVDPVNFKLLSHSLLVTL<br>ACHHPSDFTPAVHASLDKFLANVSTVLTSKYR
Hemoglobin subunit beta-A	P02077	145	16021	0.6207	0.5172	MLTAEEKAAVTGFWGKVKVDEVGAEALGRLLVVYP<br>WTQRFFEHFGDLSSADAVMNNAKVKAHGKKVLDSFS<br>NGMKHLDDLKGTFAQLSELHCDKLHVDPENFKLLGNV<br>LVVVLARHHGSEFTPLLQAEFQKVVAGVANALAHRYH
Hemoglobin subunit beta-C	P02078	142	15751	0.6056	0.5000	MPNKALITGFWSKVKVDEVGAEALGRLLVVYPWTQR<br>FFEHFGDLSSADAVLGNAKVKAHGKKVLDSFSNGVQH<br>LDDLKGTFAELSELHCDKLHVDPENFRLLGNVLVIVLA<br>RHFGKEFTPELQAEFQKVVAGVASALAHRYH
Albumin	P85295	90	10055	0.7111	0.4556	DTHKSEIAHRFNDLGRHPEYAVSVLLRHLVDEPQNLIK<br>KHGEYGFQNALIVRXXXKAPQVSTPTLVEISRKQTALV<br>ELLKLVASTQAALA

Table S2 Characteristics of the enzymes used for *in silico* analysis

Category	EINECS	Name
Dipeptidases	EC 3.4.13.9	Xaa-Pro dipeptidase
EC 3.4.13.-		
Serine Proteases	EC 3.4.21.1	Chymotrypsin (A)
EC 3.4.21.-	EC 3.4.21.19	V-8 protease (pH = 4/7.8)
	EC 3.4.21.2	Chymotrypsin C
	EC 3.4.21.20	Cathepsin G
	EC 3.4.21.26	Prolyl oligopeptidase
	EC 3.4.21.3	Metridin
	EC 3.4.21.36	Pancreatic elastase
	EC 3.4.21.37	Leukocyte elastase
	EC 3.4.21.39	Chymase
	EC 3.4.21.4	Trypsin
	EC 3.4.21.5	Thrombin
	EC 3.4.21.62	Subtilisin
	EC 3.4.21.64	Proteinase K
	EC 3.4.21.7	Plasmin
	EC 3.4.21.71	Pancreatic elastase II
	EC 3.4.21.82	Glutamyl endopeptidase II
	EC 3.4.21.83	Oligopeptidase B
	EC 3.4.21.96	Proteinase P1 (lactocepin)
Cysteine Proteases	EC 3.4.22.2	Papain
EC 3.4.22.-	EC 3.4.22.25	Glycyl endopeptidase
	EC 3.4.22.3	Ficin
	EC 3.4.22.32	Stem bromelain
	EC 3.4.22.53	Calpain 2
	EC 3.4.22.67	Ginger protease (zingipain)
	EC 3.4.22.8	Clostripain
Aspartic Proteases	EC 3.4.23.1	Pepsin (pH 1.3)
EC 3.4.23.-	EC 3.4.23.1	Pepsin (pH>2)
	EC 3.4.23.4	Chymosin
Metalloproteases	EC 3.4.24.27	Thermolysin
EC 3.4.24.-	EC 3.4.24.30	Coccolysin

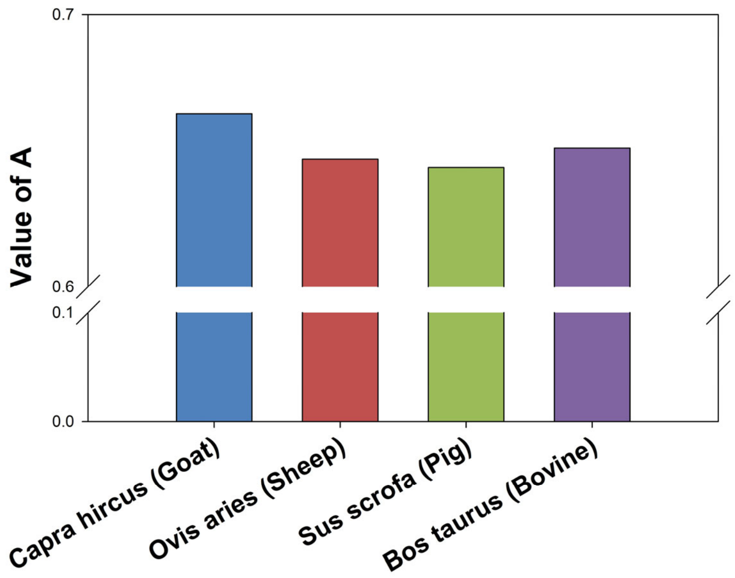

Fig. S1 The predicted A values of blood proteins in different species

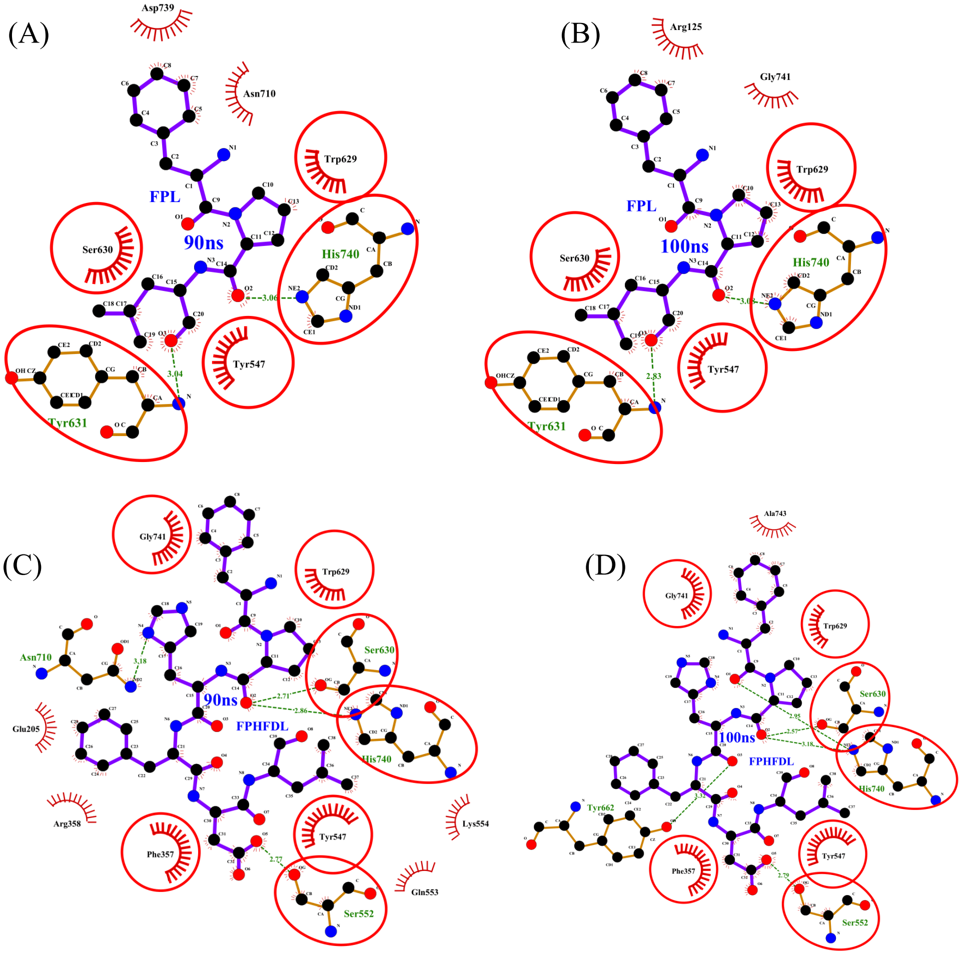

Fig. S2 Molecular docking analysis of DPP-IV enzyme (PDB ID: 1WCY) with synthetic peptides FPL and FPHFDL. (A) ligand interaction diagram of FPL-DPP-IV complex in molecular docking. (B) ligand interaction diagram of FPL-DPP-IV complex in molecular dynamics simulation. (C) ligand interaction diagram of FPHFDL-DPP-IV complex in molecular docking. (D) ligand interaction diagram of FPHFDL-DPP-IV complex in molecular dynamics simulation. Note: Hydrogen bonds are indicated by green dashed lines with distance values. Hydrophobic interactions are represented by red spoke arcs (non-ligand residues).
